# Prevalence of high-risk human papillomavirus among women in two English-speaking Caribbean countries

**DOI:** 10.26633/RPSP.2017.41

**Published:** 2017-04-14

**Authors:** Glennis Andall-Brereton, Eulynis Brown, Sherian Slater, Yvette Holder, Silvana Luciani, Merle Lewis, Beryl Irons

**Affiliations:** 1 Caribbean Public Health Agency Caribbean Public Health Agency Port-of-Spain Trinidad and Tobago Caribbean Public Health Agency, Port-of-Spain, Trinidad and Tobago.; 2 Ministry of Health Ministry of Health Saint Kitts and Nevis Ministry of Health, Saint Kitts and Nevis, Basseterre, Saint Kitts and Nevis.; 3 Ministry of Health, Wellness and the Environment Ministry of Health, Wellness and the Environment Ministry of Health, Wellness and the Environment, Saint Vincent and the Grenadines, Kingstown, Saint Vincent and the Grenadines.; 4 Office of Eastern Caribbean Coordination Pan American Health Organization/World Health Organization Barbados Office of Eastern Caribbean Coordination, Pan American Health Organization/World Health Organization, Bridgetown, Barbados.; 5 Pan American Health Organization Pan American Health Organization Washington United States of America Pan American Health Organization, Washington, D.C., United States of America.

**Keywords:** Papilloma, prevalence, uterine cervical diseases, Caribbean Region, Papiloma, prevalencia, enfermedades del cuello del útero, Región del Caribe, Papiloma, prevalência, doenças do colo do útero, Região do Caribe.

## Abstract

**Objective.:**

To characterize high-risk human papillomavirus (HPV) infections in a sample of women in two small English-speaking Caribbean countries: Saint Kitts and Nevis and Saint Vincent and the Grenadines.

**Methods.:**

Sexually active women ≥ 30 years old attending primary care health facilities participated in the study. Each participant had a gynecological examination, and two cervical specimens were collected: (1) a specimen for a Papanicolaou (Pap) test and (2) a sample of exfoliated cervical cells for HPV DNA testing, using the HPV High Risk Screen Real-TM (Sacace). High-risk HPV genotypes were assessed in 404 women in Saint Kitts and Nevis and 368 women in Saint Vincent and the Grenadines.

**Results.:**

High-risk HPV was detected in 102 of 404 (25.2%) in Saint Kitts and Nevis and in 109 of 368 (29.6%) in Saint Vincent and the Grenadines. High-risk HPV genotypes 52, 35, 51, 45, and 31 were the most common high-risk types in Saint Kitts and Nevis. In Saint Vincent and the Grenadines, the most common high-risk HPV genotypes were 45, 35, 31, 18, and 51. Current age was found to be significantly associated with high-risk HPV infection in both countries. In addition, in Saint Vincent and the Grenadines, high parity (> 3 pregnancies) and having had an abnormal Pap smear were found to be independent risk factors for high-risk HPV.

**Conclusions.:**

These results contribute to the evidence on HPV prevalence for small island states of the Caribbean and support the accelerated introduction of the 9-valent HPV vaccine in the two countries and elsewhere in the English-speaking Caribbean. Use of the study’s results to guide the development of policy regarding implementation of HPV testing as the primary screening modality for older women is recommended.

## RESUMEN

**Objetivo.:**

Caracterizar la infección por el virus del papiloma humano (VPH) de alto riesgo en una muestra de mujeres de dos países pequeños del Caribe de habla inglesa: Saint Kitts y Nevis, y San Vicente y las Granadinas.

**Métodos.:**

Participaron en el estudio mujeres sexualmente activas ≥ 30 años que acudieron a centros de atención primaria. Se realizó un examen ginecológico a todas las participantes y se le tomaron dos muestras cervicouterinas: 1) una muestra para efectuar la prueba de Papanicolaou y 2) células cervicouterinas exfoliadas para efectuar la prueba de ADN del VPH, mediante la prueba de detección del VPH de alto riesgo en tiempo real (HPV High Risk Screen Real-TM, Sacace). Se evaluaron los genotipos de alto riesgo del VPH en 404 mujeres en Saint Kitts y Nevis, y en 368 mujeres en San Vicente y las Granadinas.

**Resultados.:**

Se detectó VPH de alto riesgo en 102 de 404 mujeres (25,2%) de Saint Kitts y Nevis, y en 109 de 368 (29,6%) de San Vicente y las Granadinas. Los genotipos 52, 35, 51, 45 y 31 de VPH de alto riesgo fueron los detectados con más frecuencia en Saint Kitts y Nevis. En San Vicente y las Granadinas, los más frecuentes fueron los genotipos 45, 35, 31, 18 y 51. Se observó una relación significativa entre la edad actual y la infección por el VPH de alto riesgo en los dos países. Además, en San Vicente y las Granadinas, se halló que la multiparidad (> 3 embarazos) y los resultados anómalos de la prueba de Papanicolaou son factores de riesgo independientes de la infección por el VPH de alto riesgo.

**Conclusiones.:**

Estos resultados aportan datos acerca de la prevalencia del VPH en los estados insulares pequeños del Caribe y respaldan la introducción acelerada de la vacuna nonavalente contra el VPH en los dos países y en los demás países del Caribe de habla inglesa. Se recomienda aplicar los resultados de este estudio para guiar la formulación de políticas con respecto a la incorporación de las pruebas de detección del VPH como método primario de tamizaje en las mujeres mayores.

## RESUMO

**Objetivo.:**

Caracterizar as infecções pelo papilomavírus humano (HPV) de alto risco em uma amostra de mulheres de dois países pequenos do Caribe anglófono: São Cristóvão e Nevis e São Vicente e Granadinas.

**Métodos.:**

Mulheres sexualmente ativas com ≥ 30 anos de idade atendidas em unidades básicas de saúde participaram do estudo. Cada participante foi submetida a exame ginecológico e coleta de duas amostras do colo do útero: (1) uma amostra para exame de Papanicolau e (2) uma amostra de citologia esfoliativa para teste de DNA do HPV, utilizando o kit HPV High Risk Screen Real-TM (Sacace). A presença de genótipos de HPV de alto risco foi avaliada em 404 mulheres em São Cristóvão e Nevis e 368 mulheres em São Vicente e Granadinas.

**Resultados.:**

HPV de alto risco foi detectado em 102/404 mulheres (25,2%) em São Cristóvão e Nevis e em 109/368 (29,6%) em São Vicente e Granadinas. Os genótipos 52, 35, 51, 45 e 31 do HPV foram os tipos de alto risco mais comuns em São Cristóvão e Nevis. Em São Vicente e Granadinas, os genótipos do HPV de alto risco mais comuns foram 45, 35, 31, 18 e 51. Foi constatada associação significante entre idade atual e infecção por HPV de alto risco em ambos os países. Além disso, em São Vicente e Granadinas, multiparidade (>3 gestações) e Papanicolau anormal foram fatores de risco independentes para o HPV de alto risco.

**Conclusões.:**

Esses resultados contribuem informações sobre a prevalência do HPV nas pequenas nações insulares do Caribe e apoiam a introdução acelerada da vacina nonavalente contra o HPV nestes dois países e no restante do Caribe anglófono. Recomenda-se o uso dos resultados deste estudo para orientar a formulação de políticas com relação à implementação do exame de HPV como modalidade primária de triagem para mulheres mais velhas.

Human papilloma viruses (HPVs) are very common sexually transmitted infections. Infection with high-risk HPV genotypes has been recognized as a necessary factor for the development of cervical cancer as well as cancers of the vagina, anus, vulva, penis, and oropharynx 1. In the Caribbean, cervical cancer remains a significant public health problem. It was the second cause of cancer death among women there in 2014, according to information from the regional mortality database of the Caribbean Public Health Agency (CARPHA). Data for 2012 from the GLOBOCAN project indicated that the mortality from and incidence of cervical cancer in the Caribbean were 9 and 21 per 100 000, respectively 2.

HPV genotypes 16, 18, 31, 33, 35, 39, 45, 51, 52, 56, 58, and 59 have been identified as cervical carcinogens (3, 4). Interest in identifying HPV genotypes increased with the development of prophylactic vaccines, which have been shown to prevent high-grade lesions of the cervix; precancerous lesions of the vulva, vagina, and anus; and genital warts 1. Studies of HPV prevalence have been conducted in countries throughout the world as a means of identifying the potential impact of these vaccines in specified populations 3.

In the Caribbean, such studies have been done in cancer-free populations in three English-speaking countries: Belize, Jamaica, and Trinidad and Tobago (5–9). Before this current study, no information on HPV prevalence and distribution of highrisk types was available for the smaller countries (population sizes ≤ 100 000) of the English-speaking Caribbean.

This study identified high-risk HPV infections in a sample of women in two small archipelagic countries in the English-speaking Caribbean: Saint Kitts and Nevis (2011 estimated population of 53 000) and Saint Vincent and the Grenadines (2011 estimated population of 109 903). In addition, the study sought to determine whether HPV infection is associated with sociodemographic, behavioral, and reproductive factors in the population.

The study was conducted by the Pan American Health Organization (PAHO) in collaboration with CARPHA and the Ministries of Health of Saint Kitts and Nevis and Saint Vincent and the Grenadines. Ethical approval for the study was granted by the Ministries of Health in the respective countries and by the Pan American Health Organization/World Health Organization Ethical Review Committee, based on the assurance of confidentiality of information and the potential benefit to participants.

## MATERIALS AND METHODS

### Study population

Participants for this cross-sectional study were recruited from February to November 2014. All islands in each country and all primary health care clinics on each island were included, as well as five voluntarily participating privatesector health sites (four in Saint Kitts and Nevis and one in Saint Vincent and the Grenadines), one nongovernmental organization (NGO) (Saint Vincent Planned Parenthood Clinic), and two outreach sites at garment factories in Saint Kitts and Nevis.

A convenience sample of women 30 years or older attending primary care clinics, selected gynecologists, and NGO clinics in each country were invited to participate in the study. Informed consent was required and signed by each participant. An identification number was assigned and utilized on all study documentation for each respective participant. Participants were informed that strict confidentiality would be maintained and that no identification information would be reported, but instead used only for follow-up care. All persons meeting the study criteria and giving informed consent were entered into the study until 500 women from each country were recruited. No financial compensation was provided to participants for taking part in the study.

### Data Collection

Standard operating procedures (SOPs), which included specimen collection, were developed for the study. Midwives and nursing staff were trained in interviewing techniques. The midwives were also trained in specimen collection using the SOPs.

**Questionnaire.** Each participant was interviewed privately and confidentially by a trained health staff member (usually a nurse), using a questionnaire. The questionnaire collected information on the demographic, behavioral, and reproductive characteristics of study participants as well as knowledge of and attitudes toward immunizations against vaccinepreventable diseases, HPV in particular.

**Sample collection.** Each participant had a gynecological examination done and cervical specimens collected by the trained midwives at the study sites. Specimens were collected for a Papanicolaou (Pap) test and for HPV DNA testing using the HPV High Risk Screen Real-TM (Sacace, Como, Italy) 10.

All cervical smears taken for Pap testing were immediately sprayed with a fixative prior to labeling and being packaged for transport to the laboratory. The cervical cells for HPV testing were taken from the endocervix, using a Cytobrush, which was then placed in a specimen transport medium in a 2-ml vial.

Both specimens remained at room temperature until the end of the collection day, when they were then transported on ice packs to the central laboratory in each respective country for storage at 2 to 8 °C. The specimens collected for HPV DNA testing were later batch-shipped at 2 to 8 °C from each respective study country via air courier service to the Caribbean Genetics (CARIGEN) laboratory at the Department of Basic Medical Sciences, University of the West Indies, Mona, Kingston, Jamaica, for processing.

**Sample processing.** The Pap smear tests were processed at the University of the West Indies Pathology Laboratory, using the Bethesda cytology classification system. In order to ensure high-quality cytology results, every 10th slide was reread by another cytologist, and all positive smears were reviewed by the pathologist. At CARIGEN, the specimens that were collected for HPV DNA testing were stored at 2 to 8 oC once received, and they were extracted within three days, using an automated QIAxtractor (QIAGEN) DNA extraction instrument. The final extract was either tested immediately or stored at –20 °C prior to processing. For every set of 16 samples processed on the QIAxtractor, a phosphate-buffered saline (PBS) blank was used to serve as a “contamination control.”

These specimens were tested for HPV using the HPV High Risk Screen Real-TM (Sacace), an in vitro Real Time amplification test for quantitative detection of high-risk human papillomavirus (genotypes 16, 18, 31, 33, 35, 39, 45, 51, 52, 56, 58, 59) in urogenital brushes. The test uses primers directed against regions of HPV A9 Group (16, 31, 33, 35, 52, 58); HPV A7 Group (18, 39, 45, 59); and HPV A5-A6 Group (51, 56); and with *β*-globin gene used as internal control to check for extracted DNA. Some samples with a high quantity of mucous or insufficient quantity of epithelial cells caused problems and were not processed properly on the extractor. This resulted in the internal control not being detected. Samples with a small quantity of cells resulted in a “low cell” reading. Both low-cells samples and those with high mucous content were reextracted using the DNA IQ Manual Extraction kit (Promega, Como, Italy).

DNA was amplified using the RotorGene/6000 (Corbett Research)/Rotor-Gene Q (QIAGEN) Real Time PCR cycler, according to the manufacturer’s instructions. After amplification, data from each run was exported to an Excel spreadsheet macro provided by the manufacturer that allowed for the calculation of HPV group and viral loads. Known standards and contamination controls were run with each amplification for viral load determination and PCR amplification efficiency.

The HPV typing was reported as high-risk HPV-positive with reference to the grouping (HPV A9 Group; HPV A7 Group; HPV A5-6 Group) included in the HPV High Risk Screen Real-TM (Sacace Biotechnologies) test, as HPV-negative, or as low cells. HPV typing was reported as high-risk HPVnegative if the test was negative for the aforementioned groupings. HPV typing was reported as low cells if there were insufficient epithelial cells.

With the HPV typing, the specimens that were identified as positive were typed for HPV using also the HPV High Risk Type Real-TM (Sacace). This is an in vitro Real Time amplification test for quantitative detection of high-risk human papillomavirus types 16, 18, 31, 33, 35, 39, 45, 51, 52, 56, 58, and 59 in urogenital brushes. Each vial contains primers directed against regions of each HPV type and a *β*-globin gene used as an internal control.

DNA was amplified using the Rotor-Gene/6000 (Corbett Research)/Rotor-Gene Q (QIAGEN) Real Time PCR cycler, according to manufacturer’s instructions. After amplification, data from each run was exported to an Excel spreadsheet macro provided by the manufacturer. This allowed for the identification of the individual HPV types. Known positives were run with each amplification to check for PCR amplification efficiency. A blank sample of nuclease-free water was also run with each amplification as a contamination control. The respective HPV type was reported for all the HPV types included in the HPV High Risk Type Real-TM (Sacace) test.

### Statistical analysis

The analysis was restricted to the participants whose cervical specimens were adequate for HPV DNA isolation, which was 404 in Saint Kitts and Nevis and 368 in Saint Vincent and the Grenadines. Data were analyzed using EPi Info version 7 software. Cross-tabulations were used to explore associations of the presence of high-risk HPV infection with the several demographic, behavioral, and reproductive factors and the Pap smear results. Risk ratios and chi-squared statistics were used to determine the strength of associations. Using abnormal Pap smears and the presence of high-risk HPV infection as dependent variables, multiple logistic regression analyses were then used to model the relationship of possible risk factors from among the demographic, behavioral, and reproductive variables with these outcome variables.

## RESULTS

The results presented are for the women who had cervical specimens that were satisfactory for testing for HPV DNA. The number of cervical specimens that were not satisfactory for testing (due to problems with storage and transportation) and that were thus excluded from processing and analysis was 96 of 500 (19.2%) in Saint Kitts and Nevis and 132 of 500 (26.4%) in Saint Vincent and the Grenadines.

### Characteristics of study participants tested for high-risk HPV

In Saint Kitts and Nevis, of the 404 women who were tested for high-risk HPV, 56.5% of them were between 30 and 44 years old and 43.5% were 45 or older. In Saint Vincent and the Grenadines, of the 368 women tested, 51.7% were between 30 and 44 years old and 48.3% were 45 or over. The median age of the women in Saint Kitts and Nevis was 42 years (mean = 43.7 years; range, 30 to 81 years), while in Saint Vincent and the Grenadines, the median age was 44 years (mean = 45.2 years; range, 30 to 77 years).

There were significant differences in the demographic profile of the participants from the two countries, as shown in Table 1.

### High-risk HPV genotypes

In both of the countries in the study, a little over one-quarter of those who were tested were positive for high-risk HPV: 102 of 404 (25.2%) in Saint Kitts and Nevis and 109 of 368 (29.6%) in Saint Vincent and the Grenadines.

**FIGURE 1:**
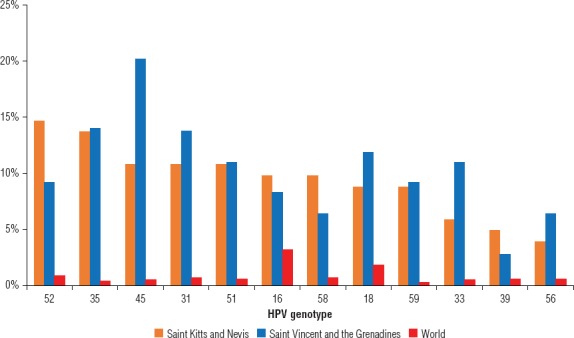
Prevalence (%) of high-risk HPV genotypes, Saint Kitts and Nevis (2015), Saint Vincent and the Grenadines (2015), and the world (2010).

***Source***: Prepared by the authors using the results of this study and the world HPV prevalence data from Bruni et al. 11.

There were 12 high-risk HPV genotypes detected in the women tested in the two countries: 16, 18, 31, 33, 35, 39, 45, 51, 52, 56, 58, and 59 (Figure 1). Figure 1 also compares the prevalence of those genotypes in the two Caribbean countries with the levels for the entire world 11.

Overall, HPV high-risk genotypes 45, 35, 31, and 52 were the most prevalent types detected in the two Caribbean countries in the study. HPV 52 and 35 were the most prevalent high-risk types in Saint Kitts and Nevis. In Saint Vincent and the Grenadines, the most prevalent types were 45, 35, and 31. HPV 16 was detected in 10 of 102 (9.8%) of the high-risk infections in Saint Kitts and Nevis and in 9 of 109 (8.3%) of those in Saint Vincent and the Grenadines. HPV 18 was identified in 9 of 102 (8.8%) of the high-risk infections in Saint Kitts and Nevis and in 13 of 109 (11.9%) of those in Saint Vincent and the Grenadines.

### High-risk HPV genotypes and cytological abnormalities

In Saint Kitts and Nevis, of the 102 women with high-risk HPV infections, 6 of them (5.9%) also had abnormal Pap smears. Of those 6, 4 of them were classified as atypical squamous cell of undetermined significance (ASCUS) and 2 were classified as low-grade squamous intraepithelial lesions (LSIL). Of those 6, 2 of them had high-risk HPV 16 infections, and none of them had an HPV 18 infection.

In Saint Vincent and the Grenadines, of the 109 women with high-risk HPV infections, 14 of them (12.8%) also had abnormal Pap smears. Of the 14 abnormal smears, 3 of them were classified as ASCUS, 5 were LSIL, and 6 were high-grade squamous intraepithelial lesions (HSIL). Of the 6 HSILs, 2 were associated with high-risk HPV 31, and 1 was associated with HPV type 16. Also, 1 HSIL was associated with HPV 18 in a multiple infection with HPV types 45, 33, and 52. The remaining 2 of the 6 HSILs were associated with HPV 52 and HPV 58. Overall in Saint Vincent and the Grenadines, 6 of the 14 (42.8%) of those with abnormal Pap smears were infected with high-risk HPV 16 or 18. Of those with abnormal smears, 4 of them (28.5%) were infected with HPV 16, and 2 of them (14.3%) with high-risk HPV 18.

**TABLE 1.** Demographic characteristics (%) of participants tested for high-risk HPV in Saint Kitts and Nevis and in Saint Vincent and the Grenadines, 2014

Characteristic	Saint Kitts and Nevis (*n* = 404)	Saint Vincent and the Grenadines (*n* = 368)	P
Marital status			< 0.01
Single	52.7	40.2	
Married	35.6	39.2	
Other	10.1	20.3	
Not stated	1.6	0.5	
Employment status			< 0.01
Employed	89.7	54.6	
Housewife	2.8	20.5	
Unemployed	5.7	24.7	
Not stated	1.8	0.2	
Education			< 0.01
Primary or less	10.9	65.6	
Secondary only	65.1	27.0	
Tertiary	7.5	2.2	
Other	15.4	4.6	
Not stated	1.2	0.7	
Gross household income			< 0.01
< 2 500 EC dollars (< US$ 932)^a^	52.1	81.0	
> 2 500 EC dollars (> US$ 932)	40.0	14.9	
Not stated	7.9	4.1	
Parity			< 0.01
0–3 pregnancies	61.1	46.7	
≥ 4 pregnancies	38.9	53.3	
No. of sexual partners in lifetime			0.01
6 or more	24.3	18.1	
3–5	44.1	46.6	
1–2	25.7	29.0	
Not stated	5.9	6.2	
Age at first coitus			< 0.01
< 17 yrs	40.3	48.9	
≥ 17 yrs	50.5	43.5	
Not stated	9.2	7.6	
Condom use at first coitus			< 0.01
No	59.9	72.8	
Yes	35.1	23.9	
Not stated	5.0	3.3	

a EC dollars = East Caribbean dollars.

In the two countries, more than a fifth of the women with high-risk HPV were infected with multiple types of high-risk HPV. This was true in Saint Kitts and Nevis for 28 of 102 women (27.4%) and in Saint Vincent and the Grenadines for 23 of 109 women (21.1%). Just over a third of the total number of multiple infections in each country included high-risk HPV 16 or 18: in Saint Kitts and Nevis, 11 of the 28 (39.3%), and, in Saint Vincent and the Grenadines, 9 of 23 (39.1%). Multiple high-risk types were identified in 2 of 6 of those with abnormal smears (1 ASCUS and 1 LSIL) in Saint Kitts and Nevis and 6 of 14 (42.8%) of those with abnormal smears (2 ASCUS, 3 LSIL, and 1 HSIL) in Saint Vincent and the Grenadines.

### High-risk HPV and risk factors

In Saint Kitts and Nevis, only current age was found to be very significantly associated with high-risk HPV infection (*P* < 0.01). In Saint Vincent and the Grenadines, in addition to current age, number of sexual partners in the lifetime (≥ 3 partners), number of pregnancies (> 3 pregnancies), and having had an abnormal Pap smear were also significantly associated with high-risk HPV status (*P* < 0.05). In Saint Kitts and Nevis, participants with abnormal Pap smears also had a high prevalence of high-risk HPV. However, no significant association could be ascertained due to the small numbers (Table 2).

Multivariate logistic regression analyses confirmed the associations and identified them as independent risk factors. In both Saint Kitts and Nevis and in Saint Vincent and the Grenadines, younger women were approximately twice as likely to have a high-risk HPV infection (risk ratio (RR) of 2.38 and of 1.85, respectively). In Saint Vincent and the Grenadines, parity > 3 pregnancies and abnormal Pap smear result were also identified as independent significant risk factors, with such women more than twice as likely to have a high-risk HPV infection (RR of 2.31 and of 2.44, respectively). The number of sexual partners, though associated, was not identified as an independent risk factor for high-risk HPV infection (Table 3).

## DISCUSSION

The study used a convenience sample, and the recruitment of study participants was done at sites associated with the provision of health care. As a consequence, women who do not readily access health care may have been excluded. This may have introduced bias into the study, so the results are not generalizable to the general population of women 30 years and older or those with other healthseeking behaviors in the two countries.

The results reported are only for the women who had cervical specimens that were satisfactory for testing for HPV DNA. A large percentage had cervical specimens that were unsatisfactory for testing: 96 of 500 (19.2%) of study participants in Saint Kitts and Nevis, and 132 of 500 (26.4%) in Saint Vincent and the Grenadines. These specimens could not be processed and were therefore excluded from the analysis. As a consequence, there is a possibility that important information may have been lost by not being able to process the unsatisfactory cervical specimens. Issues such as the need for air courier shipment service from each respective study country to Jamaica and inadequate cold storage of cervical specimens from both countries may have impacted the quality of the specimens and resulted in loss of valuable information.

**TABLE 2.** Factors associated with high-risk-HPV-positive women in Saint Kitts and Nevis and Saint Vincent and the Grenadines, 2014

Variable	Saint Kitts and Nevis				Saint Vincent and the Grenadines			
	Subpopulation (n)	HPV prevalence		Chisquared	P	Subpopulation (n)	HPV prevalence		Chisquared	P
		n	%				n	%		
Age group				12.7	< 0.01				4.51	0.03
30-44 years	228	73	32.0			188	65	34.6		
≥ 45	176	29	16.5			180	44	24.4		
Age at first coitus				0.22	0.64				0.14	0.70
< 17 years	162	43	26.5			180	54	30.0		
≥ 17 years	205	50	24.4			160	45	28.1		
Contraceptive use at first coitus^a^				0.00	0.97				2.52	0.11
Yes	142	36	25.4			88	32	36.4		
No	262	66	25.2			280	77	27.5		
Number of sexual partners in lifetime				4.15	0.13				8.44	0.01
≥ 6	98	31	31.6			70	27	38.6		
3–5	178	44	24.7			180	57	31.7		
1–2	104	20	19.2			112	22	19.6		
Number of pregnancies				0.15	0.70				5.17	0.02
0–3	247	64	25.9			172	41	23.8		
≥ 4	157	38	24.2			196	68	34.7		
Abnormal Pap smear				1.79	0.18				7.86	0.01
Yes	19	6	40.0			26	14	53.8		
No	39	96	24.7			342	95	27.8			

***Source:*** Prepared by authors using the results of this study.

a Contraceptive use = use of condom or diaphragm as barrier method capable of reducing the risk of HPV infection.

The study sites used were in both the public and private health sector. However, the number of participants recruited from the latter were small and therefore did not yield enough data to detect differences in demographic, behavioral, and reproductive factors or HPV prevalence between the participants seen in public and private health care settings.

To our knowledge, this is the first report on HPV prevalence and HPV genotypes done in any of the islands of the Organization of Eastern Caribbean States (OECS). The prevalence of high-risk HPV infections in Saint Kitts and Nevis (25.2%) and in Saint Vincent and the Grenadines (29.6%) was similar to the prevalence of high-risk HPV infections (20.3%) found in a study done in Tobago by Ragin et al. 8. However, it was less than the prevalence of high-risk HPV infections found in Trinidad (65.8%) (9) and in Jamaica (34.9%) 6. These differences may be due to the different HPV testing methods used in these studies as well as differences in the age groupings studied.

**TABLE 3.** Independent risk factors for high-risk HPV in women in Saint Kitts and Nevis and in Saint Vincent and the Grenadines, 2014, with risk ratios and 95% confidence intervals (CIs)

Risk factor or variable	Saint Kitts and Nevis			Saint Vincent and the Grenadines		
	Risk ratio	95% CI	P	Risk ratio	95% CI	P
High parity (> 3 pregnancies)	1.09	0.63–1.87	0.76	2.31	1.30–4.14	< 0.01
No barrier contraception at first coitus (condom or diaphragm)	1.14	0.67–1.93	0.63	0.56	0.30–1.03	0.06
Age group (30–44 years)	2.38	1.35–4.17	< 0.01	1.85	1.06–3.33	0.03
Age at first coitus (> 17 years)	1.07	0.63–1.85	0.78	1.03	0.61–1.74	0.92.
No. of sexual partners in lifetime (3–5)	1.27	0.70–2.27	0.44	1.20	0.64–2.27	0.56
No. of sexual partners in lifetime (> 5)	1.75	0.84–3.70	0.14	2.13	1.00–4.55	0.05
Abnormal Pap smear	1.87	0.59–5.96	0.28	2.44	1.02–5.84	0.04

***Source:*** Prepared by authors using results from this study.

**TABLE 4.** Prevalence (%) of high-risk HPV genotypes in various Caribbean countries, according to this study and other research

Genotype	Saint Kitts and Nevis (this study)	Saint Vincent and the Grenadines (this study)	Jamaica (Lewis-Bellet al. 2013 (6))	Jamaica (Watt et al. 2009 (7))	Trinidad (Andall-Brereton et al. 2011 (9))	Tobago (Ragin et al. 2007 (8))	Belize (Cathro et al. 2009 (5))	Caribbean (Tobago & Jamaica) (Ragin et al. 2009 (12))
16	9.8	8.3	6.2	18.4	9.5	6.7	2.8	13
18	8.8	11.9	4.3	14.5	8.6	2.7	2.2	14
31	10.8	13.8	2.7	7.2	3.3	5.3	1.3	7
33	5.9	11.0	1.4	1.4	4.0	2.7	1.1	2
35	13.7	14.0	6.0	15.1	1.3	6.7	1.1	17
39	4.9	2.8	2.8	1.9	0.6	5.3	0.9	5
45	10.8	20.2	3.5	21.7	2.0	10.7	1.3	26
51	10.8	11.0	3.3	11.1	2.0	2.7	0.7	18
52	14.7	9.2	3.6	12.0	12.7	6.7	1.5	14
53	NRa	NR	NR	NR	3.3	NR	0.4	17
56	3.9	6.4	2.2	2.9	0.6	2.7	1.7	3
58	9.8	6.4	5.4	18.8	7.9	2.7	1.1	3
59	8.8	9.2	2.2	4.3	2.0	4.0	0.7	2
66	NAb	NA	4.2	NR	10.3	NR	1.3	23
68	NA	NA	2.3	3.9	NR	NR	1.3	4
73	NA	NA	NR	NR	1.3	5.3	0.2	9

***Source:*** Prepared by authors using results from this study and data from published HPV prevalence studies done in the English-speaking Caribbean.

a NR = not reported.

b NA = not available.

The distribution of the most prevalent (> 10%) high-risk HPV types showed similarities in Saint Kitts and Nevis and Saint Vincent and the Grenadines. HPV 52, 35, 51, 45, and 31 were the most prevalent in Saint Kitts and Nevis, while

HPV 45, 35, 31, 18, and 51 were the most common in Saint Vincent and the Grenadines. Table 4 compares the high-risk genotypes identified in this study with those identified from research in other Caribbean countries. HPV 45 was the most prevalent high-risk HPV type found in the Tobago study (8) and in the study done in Jamaica by Watt et al. 7. HPV 52 was the most prevalent highrisk type in the study done in Trinidad 9. Other high-risk types prevalent in the Trinidad study were HPV 66, 16, 18, and 58. Except for HPV 35, which ranked second, the ranking of the highrisk genotypes prevalent in this study differed from those identified in the Jamaica study done by Lewis-Bell et al. 6. Differences in the age groups studied may have contributed to the differences in prevalence of the genotypes. Other common high-risk types reported in the Caribbean include HPV 66, 51, 35, and 53 12.

HPV 16 and 18 were *not* the most prevalent high-risk types in the two countries in this study, and other studies have shown that same pattern is also true for other nations in the Caribbean 5. In this study, lower prevalence levels of infection with HPV 16 were also seen in the abnormal smears classified as HSIL. This study highlights differences between the prevalent high-risk genotypes in the Caribbean and those seen in United States and European populations of women with normal cytology as well as in high grade lesions (13-15). However, this study is not able to report on whether HPV 16 and 18 are the most prevalent in women with cervical cancer, as has been reported in other international studies 3.

The majority of women in this study (94% in Saint Kitts and Nevis and 87% in Saint Vincent and the Grenadines) infected with high-risk HPV had normal cytology. This has also been shown in other HPV studies done in the Caribbean (5, 6, 11).

The contribution of HPV genotypes 16, 18, 31, 33, 35, 45, 51, 52, 56, 58, and 59 to cervical disease is well established (3, 4). These high-risk genotypes are common and have been shown to account for varying levels of cervical disease in different regions of the world 14. The 9-valent HPV vaccine is expected to provide protection against HPV 31, 33, 45, 52, 58, 6, 11, 16, and 18 and thus to prevent almost 90% of invasive cervical cancers worldwide 1. Additionally, the 9-valent vaccine is anticipated to reduce other HPV-related cancers (1, 4). The most prevalent high-risk HPV genotypes in Saint Kitts and Nevis were HPV 52, 35, 51, 45, and 31. In Saint Vincent and the Grenadines, they were HPV 45, 35, 31, 18, and 51. Therefore, the most-prevalent high-risk HPV genotypes identified in Saint Kitts and Nevis and Saint Vincent and the Grenadines are covered by the 9-valent vaccine (HPV 31, 33, 45, 52, 58, 6, 11, 16, 18), except for HPV 35. Consequently, the 9-valent vaccine holds great promise for reducing cervical disease in Saint Kitts and Nevis and Saint Vincent and the Grenadines. Further research is needed to develop a vaccine against HPV 35, which had the second highest prevalence in both countries.

Co-infection with other high-risk HPV types was identified in just over a fifth of the women with high-risk HPV in the two countries: 27.4% in Saint Kitts and Nevis, and 21.1% in Saint Vincent and the Grenadines. A similar percentage (24.4%) of multiple HPV infections (low risk and high risk) was identified in the Jamaica study 6. An even higher percentage (30.2%) of multiple infections was identified in Trinidad 9. In the two countries in this study, participants with abnormal Pap smears had more infections with multiple high-risk HPV types. Therefore, this study reinforces the reports of the high prevalence of infection with multiple HPV types in other Caribbean countries.

In this study, younger age (30–44 years) was identified as a risk factor associated with high-risk HPV in both countries. Other studies, done in Belize and Trinidad, have identified the highest prevalence of HPV infections in the younger age groups (5, 9), with a relatively sharp drop after age 44 5. Other risk factors associated with high risk in Saint Vincent and the Grenadines were high parity (> 3 pregnancies), having had ≥ 3 sexual partners in the lifetime, and having had an abnormal Pap smear. Having ≥ 3 pregnancies was also found to be associated with HPV infection in Trinidad 9.

### Conclusions

This is the only known study of HPV prevalence and associated risk factors done in the countries of the Organization of Eastern Caribbean States. These results contribute to the evidence on HPV prevalence for small island states and other nations of the English-speaking Caribbean.

This research supports the accelerated introduction of HPV vaccines in the immunization schedule of the two study countries, since most of the circulating HPV genotypes are in the 9-valent HPV vaccine. The two countries could use the school health system to implement this vaccination program. In addition, continued research to develop a HPV vaccine for protection against HPV 35, the second most prevalent high-risk HPV genotype in both countries, is strongly recommended.

The study’s results can also be used to guide decision-making regarding the introduction of the 9-valent HPV vaccine and the use of HPV testing as the primary screening modality for older women in other English-speaking Caribbean countries. It should be possible to conduct the processing and testing of HPV specimens in-country so as to reduce possible negative outcomes relating to the quality of the specimens.

### Acknowledgments

The authors wish to thank Oswaldo Benitez, who assisted with the development of the proposal; Wendel Guthrie (Jamaica) and Derrek Jeffers (Saint Kitts and Nevis) for providing clinical updates for the nurses in primary health care and NGO facilities that collected cervical specimens for the HPV study; and the private health facilities and nongovernmental organizations that collaborated and participated in the study. For the processing of the cervical specimens, we recognize the Caribbean Genetics (CARIGEN) laboratory, Department of Basic Medical Sciences, University of the West Indies, Mona, Kingston, Jamaica, as well as the Pathology Department at that same university. Special thanks also go to the Ministries of Health of Saint Kitts and Nevis and Saint Vincent and the Grenadines and their respective steering committees for collaboration and execution of the study through nurses and doctors collecting the data and cervical specimens. In Saint Kitts and Nevis, the steering committee included: Patrick Martin, Judy Nesbett, Eulynis Brown, Jenevie Daniels, Rhonda Lowry-Robinson, John Essein, Candace Gumbs, Clester Roberts, Derrek Jeffers, and Patrice Lawrence-Williams. In Saint Vincent and the Grenadines, the steering committee included: Simone Keizer-Beach, Sherian Slater, Camille Nichols, Ferosa Roache, Arlitha Scott, Ronald Child, Claudette Williams, and Anneke Wilson.

### Funding

The study was supported by a grant from the Albert B. Sabin Vaccine Institute (Washington, D.C., United States of America). This organization was not involved in the study development or its execution.

### Disclaimer

Authors hold sole responsibility for the views expressed in the manuscript, which may not necessarily reflect the opinion or policy of the *RPSP/ PAJPH* or PAHO.
